# Structural and Energetic Effects of A_2A_ Adenosine Receptor Mutations on Agonist and Antagonist Binding

**DOI:** 10.1371/journal.pone.0108492

**Published:** 2014-10-06

**Authors:** Henrik Keränen, Hugo Gutiérrez-de-Terán, Johan Åqvist

**Affiliations:** Department of Cell and Molecular Biology, Uppsala University, Biomedical Center, Uppsala, Sweden; University of Bologna & Italian Institute of Technology, Italy

## Abstract

To predict structural and energetic effects of point mutations on ligand binding is of considerable interest in biochemistry and pharmacology. This is not only useful in connection with site-directed mutagenesis experiments, but could also allow interpretation and prediction of individual responses to drug treatment. For G-protein coupled receptors systematic mutagenesis has provided the major part of functional data as structural information until recently has been very limited. For the pharmacologically important A_2A_ adenosine receptor, extensive site-directed mutagenesis data on agonist and antagonist binding is available and crystal structures of both types of complexes have been determined. Here, we employ a computational strategy, based on molecular dynamics free energy simulations, to rationalize and interpret available alanine-scanning experiments for both agonist and antagonist binding to this receptor. These computer simulations show excellent agreement with the experimental data and, most importantly, reveal the molecular details behind the observed effects which are often not immediately evident from the crystal structures. The work further provides a distinct validation of the computational strategy used to assess effects of point-mutations on ligand binding. It also highlights the importance of considering not only protein-ligand interactions but also those mediated by solvent water molecules, in ligand design projects.

## Introduction

Modulation of signal transduction across the cellular membrane is one of the main targets for pharmaceutical research. Most cellular signaling in eukaryotes is mediated by receptors belonging to the superfamily of G-protein coupled receptors (GPCRs), which have been consequently identified as targets for about 30% of all marketed drugs [1]. Progress in the characterization of the GPCRs has been impressive in the last decades. It encompasses breakthroughs in molecular biology and cloning, biochemical elucidation of signaling mechanisms and pathways, pharmacology and, more recently, 3D structure determination [2]. With the current capacity to generate high resolution crystal structures, the field of computer-aided structure-based drug design is becoming increasingly relevant [3]. Computational methods that have traditionally been applied to other systems can thus now be adapted for investigation of GPCR-ligand interactions. In addition, mutagenesis studies have been performed on GPCRs for more than 30 years to explore which amino acid residues are important for binding of different ligands [4]. This data can be combined with novel structural information and structure-activity relationships for series of ligands, providing an ideal situation for characterizing ligand binding through computational modeling.

The A_2A_ adenosine receptor (A_2A_AR) is one of the best-characterized GPCRs. The first crystal structure of inactive A_2A_AR was solved in complex with the inverse agonist ZM241385 [5]. This was followed by more structures of the inactive receptor in complex with several antagonists or inverse agonists [6]–[9]. In addition, active-like forms of this receptor (herein referred to as A_2A_AR*) were crystallized in complex with various agonists, all of them derived from the adenosine chemical scaffold [10], [11]. Although none of these active-like forms included the intracellular partner (the G-protein or a mimic), the conformational changes associated with receptor activation in the A_2A_AR*-agonist structures were in agreement with those observed for the fully active conformations of the β2 adrenoceptor [12] and rhodopsin [13]. The A_2A_AR and A_2A_AR* structures have become targets for several computational studies to evaluate methods and protocols [14]–[18]. These efforts include virtual screening where several new chemical scaffolds found were identified as either agonist or antagonist ligands for this receptor [7], [19]–[24].

**Figure 1:**
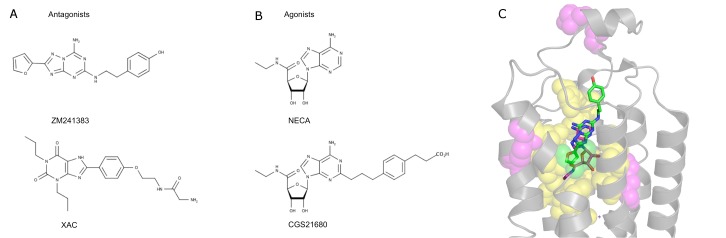
Chemical structures of ligands and overview of the A_2A_AR orthosteric binding site. Structures of the antagonist ZM241385 (A) and the agonist NECA (B) are depicted together with compounds used as radioligands in the experimental site-directed mutagenesis considered in this work (see Tables 1, 2). (C) ZM241385 (green sticks) and NECA (magenta sticks) overlaid in the orthosteric binding site [9], [10]. Receptor residues examined by alanine scanning are shown as spheres for antagonist binding (green), agonist binding (magenta) or both agonist and antagonist binding (yellow).

The A_2A_AR has also been extensively explored with alanine scanning experiments and radioligand binding assays for both agonist and antagonist binding [25]–[30]. In these experiments, the binding properties of the wild-type (wt) and mutant receptors are first characterized by means of saturation assays with a reference radioligand, which can either be an agonist or an antagonist. Thereafter, the binding affinities of a series of agonists and/or antagonists are measured in terms of their ability to competitively displace the radioligand, yielding ligand affinity ratios between the receptor variants (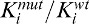). Several of the reference radioligands and competitive ligands evaluated in these assays have been co-crystallized with A_2A_AR or A_2A_AR*, which provides valuable information for linking available pharmacological and structural data [5], [10]. Figure 1 shows the chemical structures of the radioligands and competitive ligands used in the experimental mutagenesis studies considered herein.

We have recently developed an efficient free energy calculation scheme to model alanine scanning mutagenesis and compute ligand binding free energies for receptor mutants [31]. The approach was originally applied to investigate ligand binding to a homology model of the Y1 neuropeptide receptor with alanine scanning data for thirteen receptor positions, involving seven different types of sidechain mutations. Moreover, the same scheme was used to evaluate the structure-activity relationships for a series of eight antagonist ligands. The binding affinities for this series differ by over a factor of 1000 and were correctly reproduced by the simulations, while an incorrect ligand conformation was shown to cause complete loss of correlation with the experimental data [31]. In the present work, we apply this protocol to explore the available alanine scanning data of agonist and antagonist binding to the A_2A_AR, taking advantage of the structural information available for this system. The protocol is also extended to cover several amino acid mutations to alanine that were not previously considered. An excellent agreement with experimental results is achieved and the computer simulations provide key information regarding the molecular mechanism of both agonist and antagonist binding.

## Materials and Methods

### Preparation of A_2A_AR active and inactive structures

The high resolution crystal structure of human A_2A_AR (PDB code 4EIY [9]) with the antagonist ZM241385 was used as a starting point for the A_2A_AR-antagonist simulations, after minimal model refinements. This includes deletion of the engineered BRIL fusion protein, modeling of the missing extracellular loop regions (C-terminal fragment of EL2 and most of EL3) and addition of protons as described elsewhere [32]. However, the available crystal structure of NECA in complex with thermostabilized active-like form of the receptor (PDB code 2YDV [10]) was not suitable as a direct starting point for our calculations because it contains some stabilizing mutations of residues that we aimed to study, and a deformed helix VII backbone due to a *cis-*proline in the NPxxY motif. Therefore, the initial A_2A_AR*-NECA complex was obtained by combining structural information from the A_2A_AR-ZM241385 structure (PDB code 4EIY) and the two active-state A_2A_AR* structures in complex with the agonists UK432097 (PDB code 3QAK [11]) and NECA (PDB code 2YDV [10]), as recently reported [32]. Briefly, a morphing process was applied between the initial (A_2A_AR-ZM241385) and target receptor structures (A_2A_AR*-UK432097), followed by superposition of the A_2A_AR*-NECA complex where the ligand and key water molecules were retained [10]. For simulation of the A_2A_AR*-CGS241385 complex, the agonist was built from the crystal structure of the chemically similar NECA compound by superimposing the common scaffold. The initial orientation of the C2 substituent was then guided by the structure of the A_2A_AR-ZM241385 complex which has a similar moiety attached to the equivalent carbon. Standard residue sequence numbering for the human A_2A_AR is used herein, with superscripts according to the GPCR-specific position numbering based on TM helix conservation [33].

### Membrane insertion and system equilibration

The two starting structures were treated with the membrane insertion and equilibration protocol implemented in the GPCR-ModSim web-server [34]. Briefly, the system is embedded in a pre-equilibrated POPC (1- palmitoyl-2-oleoyl phosphatidylcholine) membrane model so that the TM bundle is parallel to the vertical axis of the membrane. The system is then soaked with bulk water and inserted into a hexagonal prism-shaped box of approximately 50.000 atoms (∼74% solvent molecules, ∼15% lipids, and ∼11% protein and ligand atoms). This is followed by energy minimization and a 5 ns partially restrained MD equilibration with periodic boundary conditions (PBC) using GROMACS4.0.5 [35]. This protocol consists of a first phase of 2.5 ns where positional restraints on the protein and ligand atoms are gradually released, followed by 2.5 ns where positional restraints are only applied to the protein α-carbons [36]. The standard OPLS all-atom (OPLS-AA) force-field [37], [38] was used for the protein, with ligand parameters generated by the Schrödinger utility ffld_server [39] and membrane parameters taken from the Berger united-atom model for the POPC lipids [40].

### Molecular dynamics simulation parameters

An extended ligand binding site region was extracted from the equilibrated PBC simulation system described above by including all protein atoms, POPC and water molecules within 36 Å of the designated sphere center. This center was located at the C5 of triazolotriazine connected to the aminoethylphenol moiety of ZM241385 in the A_2A_AR- ZM241385 system and the corresponding position was also used for A_2A_AR*-NECA. This reduced model of the orthosteric binding site was then used to perform MD simulations with spherical boundary conditions using the software Q [41]. With the sphere centers described above, two alternative simulation systems were constructed with radii of 25 and 34 Å, respectively. The force field scheme used was the same as in the previous MD equilibration. All ionizable residues within 5 Å of the sphere boundaries were neutralized to account for long-range dielectric screening, while protonation states of ionizable residues within the inner spherical system were assigned according to the most probable charge state at pH 7. Particular attention was paid to histidine residues, which were modeled as neutral with the proton on Nδ in all cases except His250^6.52^, His155^ECL2^ (protonated at Nε) and His264^ECL3^ (positively charged). One difference between the setup of A_2A_AR and A_2A_AR* simulations was located in the sodium binding site recently revealed in the high-resolution structure of A_2A_AR-ZM241385. The conserved residue Asp55^2.50^ was modeled as charged in the inactive state where it coordinates a sodium ion [9]. Conversely, we modeled a neutral (protonated) Asp55^2.50^ in the A_2A_AR*, to counterbalance the lack of the sodium ion in the active-like state due to the inward movement of TM7 [32]. Finally, the inactive crystal structure of A_2A_AR includes two rotamers of Thr88^3.36^ and both were considered in parallel simulations.

All atoms outside the simulation sphere were tightly restrained to their initial coordinates with a force constant of 200 kcal mol^−1 ^Å^−2^ and excluded from non-bonded interactions. A restraint of 20 kcal mol^−1 ^Å^−2^ to the initial coordinates was applied to solute atoms within the outer 3 Å shell of the spherical systems. Water molecules at the sphere surface were subjected to radial and polarization restraints according to the SCAAS model [41], [42]. Non-bonded interactions were truncated at a 10 Å cutoff, beyond which long range electrostatic interactions were treated with the local reaction field method [43] except for the atoms of the particular sidechain undergoing the alchemical modification, for which no cut-off was used. Solvent bonds and angles were constrained using the SHAKE algorithm [44]. An additional 0.61 ns equilibration phase involved stepwise heating of the spherical system from 0.1 to 298 K concomitant with release of heavy atom positional restraints (from an initial force constant of 25 kcal/mol/Å^2^). The apo structures were produced by removing the respective ligand and filling the created cavity with waters, thereafter the same equilibration procedure was applied. All production runs were done at 298 K using a separate thermal bath coupling for solute and solvent. The MD free energy calculation production phase for each holo and apo system involved a total simulation time of 3.5–5.6 ns, depending on the amino acid mutation explored, using a 1 fs MD time step. Each simulation was repeated seven times with different initial (velocity) conditions.

### Free energy calculations

We applied a recently developed free energy perturbation (FEP) protocol for amino acid mutations [31]. In short, the given mutation is divided into a series of smaller subperturbations to allow a smoother transformation between the end-states. As shown earlier, this results in a computational protocol with increased accuracy and convergence as compared to the standard free energy perturbation schemes with fewer intermediate states [31]. Each subperturbation is divided into 51 FEP windows (λ-steps), where each such window is sampled for 30–40 ps. The subperturbations are defined by atom-groups that are annihilated in succession, depending on their topological distance to the protein backbone, and undergo one perturbation at a time. During the annihilation of a residue each atom-group will undergo three consecutive transformations *i*) annihilation of partial charges, *ii*) transformation of the regular van der Waals (Lennard-Jones) potential to a soft-core potential [31] to prevent singularities and *iii*) annihilation of the soft-core potential. In each subperturbation different combinations of atom-groups can be in any of the above three stages. In the last subperturbation, annihilation of atoms directly linked to Cβ is accompanied by the creation of the final alanine Cβhydrogen atom. As an example, the procedure yields about 360 separate FEP windows for mutation of methionine and histidine into alanine.

## Results

Human A_2A_AR has been thoroughly studied by alanine scanning, which is reflected by the 38 single alanine mutations indexed in the GPCRDB database [45]. From those, and also including two additional mutations not indexed in this database, we extracted fourteen mutations for which there is experimental binding affinity data for the antagonist ZM241385 and seventeen mutations with corresponding data for the agonist NECA. Thirteen of these positions have been tested with both ligands (Figure 1C), and overall the data shows a wide spectrum of experimental ligand binding affinity changes (Tables 1, 2).

**Table 1:** Calculated and experimental ZM241385 relative binding free energies for A_2A_AR mutants.

Mutant	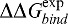 a	Radioligand	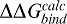
V84^3.32^A	NB^b^ (>1.4) [30]	[^3^H]XAC	3.7±0.4
T88^3.36^A	0.9±0.5 [27]	[^3^H]XAC	0.8±0.5
Q89^3.37^A	−0.6±0.1 [27]	[^3^H]XAC	−0.8±0.4
S90^3.38^A	−0.2±0.1 [27]	[^3^H]XAC	0.2±0.4^c^
S91^3.39^A	0.4±0.1 [27]	[^3^H]XAC	−0.1±1.0^c^
F168^5.29^A	NB^b^ (>1.4) [25]	[^3^H]ZM241385	2.2±0.4
E169^5.30^A	NB^b^ (>1.5) [28]	[^3^H]XAC	2.7±1.5
M177^5.38^A	1.2±0.2 [25]	[^3^H]ZM241385	1.2±0.8
L249^6.51^A	NB^b^ (>1.4) [25]	[^3^H]ZM241385	5.7±0.7
H250^6.52^A	NB^b^ (>2.3) [29]	[^3^H]XAC	2.8±0.7
N253^6.55^A	NB^b^ (>2.3) [29]	[^3^H]XAC	4.5±0.5
I274^7.39^A	NB^b^ (>1.4) [29]	[^3^H]XAC	5.4±1.0
S277^7.42^A	−0.2±0.2 [29](XAC)^d^−0.1±0.2 (CGS15943)	[^3^H]XAC	0.3±0.3
H278^7.43^A	NB (>2.3) [29]	[^3^H]XAC	3.5±1.5

a Experimental relative binding free energies (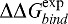) calculated from *K*_i_ values as 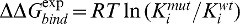.

b NB = non-detectable radioligand binding. The value corresponding to the experimental detection threshold is indicated within parentheses.

c A Simulation sphere of 34 Å radius was used, since the mutated position is outside the boundaries of the default 25 Å sphere.

d Experimental data is only available for the antagonists XAC and CGS15943.

**Table 2:** Calculated and experimental NECA relative binding free energies for A_2A_AR mutants.

Mutant	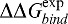 a	Radioligand	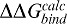
V84^3.32^A	NB^b^ (>1.4) [30]	[^3^H]NECA	4.7±1.0
T88^3.36^A	2.6±0.2 [27]	[^3^H]XAC^c^	4.7±0.2
Q89^3.37^A	−1.6±0.1 [27]	[^3^H]CGS21680	−0.9±0.8
S90^3.38^A	−0.9±0.0 [27]	[^3^H]CGS21680	−0.2±0.2
S91^3.39^A	0.2±0.0 [27]	[^3^H]CGS21680	0.6±0.4
E151^5.12^A	NB^b^ (>1.4) [28]	[^3^H]CGS21680	1.1±0.6^d^
E161^5.22^A	0.4±0.2 [28]	[^3^H]CGS21680	0.5±1.1^d^
F168^5.29^A	NB^b^ (>1.4) [25]	[^3^H] ZM241385	4.7±1.0
E169^5.30^A	NB^b^ (>2.7) [28]	[^3^H]CGS21680	4.5±1.8
M177^5.38^A	−0.2±0.5 [25]	[^3^H] ZM241385	2.6±0.8
F180^5.41^A	0.5±0.3 [29]	[^3^H]CGS21680	−0.8±0.3
H250^6.52^A	NB^b^ (>2.3) [29]	[^3^H]CGS21680	1.5±0.7
N253^6.55^A	NB^b^ (>2.3) [29]	[^3^H]CGS21680	2.6±0.7
C254^6.56^A	−0.1±0.5 [29]	[^3^H]CGS21680	0.2±0.5^d^
I274^7.39^A	NB^b^ (>2.3) [29]	[^3^H]CGS21680	3.2±0.8
S277^7.42^A	3.5±0.2 [29]	[^3^H]XAC^c^	0.5±0.4
H278^7.43^A	NB^b^ (>2.3) [29]	[^3^H]CGS21680	2.6±1.8

a Experimental relative binding free energies (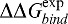) calculated from *K*_i_ values as 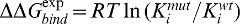.

b NB = non-detectable radioligand binding. The value corresponding to the experimental detection threshold is indicated with in parentheses.

c Non-detectable binding for the agonist radioligand CGS21680.

d A simulation sphere of 34 Å radius was used, since the mutated position is outside the boundaries of the default 25 Å sphere.

Starting from the available structures of the hA_2A_AR and hA_2A_AR* [9]–[11], we set up the A_2A_AR-ZM241385 and A_2A_AR*-NECA systems, which were subsequently equilibrated in an atomistic model of the membrane. Thereafter we created the spherical systems for MD sampling and free energy calculations, which were centered on the ligand binding site and large enough to include all positions mutated to alanine (Figure 1C). In order to rule out the possible dependency of the results on the sphere radius, most mutations were simulated with two different sphere sizes (25 Å and 34 Å radii). Since, as expected, higher precision is generally obtained with the smaller system (see below), we will focus on the results obtained with the 25 Å radius sphere except for those distal mutations that require the larger simulation system (S90^3.38^A, S91^3.39^A for A_2A_AR-ZM241385 and E151^5.12^A, E161^5.22^A, C254^6.56^A for A_2A_AR*-NECA). The results obtained with the larger sphere size are generally very similar (Tables S1, S2, Supporting Information).

Each MD simulation was replicated seven times in both the holo and apo states, yielding a total simulation time of 50–80 ns per mutation. We assessed the stability of the ligands in the binding site of the WT receptor from the average structures of all equilibrated initial WT complexes preceding the mutation calculations, which corresponds to roughly 50 ns of unrestrained simulation for each receptor conformation. The ligand and binding site (defined as residues within 5 Å from respective ligand) heavy atom RMSD was then calculated with respect to the corresponding starting structure. For the A_2A_AR-antagonist complex, these values were RMSD_binding site = _0.6 Å and RMSD_ligand = _3.2 Å. It should, however, be noted here that the phenol moiety of ZM241385 shows considerable variability between the experimental structures [5], [6], [8], [9] with an RMSD_ligand_ of 5.2 Å. If this group is omitted from the calculation the RMSD_ligand_ value between the average MD structure and 4EIY drops to 0.4 Å. For the A_2A_AR*-agonist complex with NECA the corresponding values were equally low with RMSD_binding site = _0.5 Å and RMSD_ligand = _0.8 Å.

The application of our smooth FEP protocol for sidechain annihilation resulted in accurate predictions of the effects of the mutations on ligand binding affinities (Tables 1, 2) and good convergence of the free energies. The precision was assessed by calculating the pooled standard errors of the mean (s.e.m.) based on the fourteen independent trajectories of the apo and holo forms. This yields an overall average error of 0.7 kcal/mol for the two systems, A_2A_AR-ZM241385 (Table 1) and A_2A_AR*-NECA (Table 2). This value increases to 1.0 kcal/mol for the results solely based on the larger 34 Å sphere (Tables S1, S2), which is indicative of larger conformational fluctuations when a larger part of the receptor is sampled, but the calculated free energies are generally very similar. The convergence was further estimated as the average hysteresis value, defined as the difference between carrying out the free energy calculations in the forward and the reverse direction of the transformations. In all cases, this hysteresis was below 0.5 kcal/mol, with an average value of 0.2 kcal/mol for all the transformations performed.

### Binding of the antagonist ZM241385

**Figure 2:**
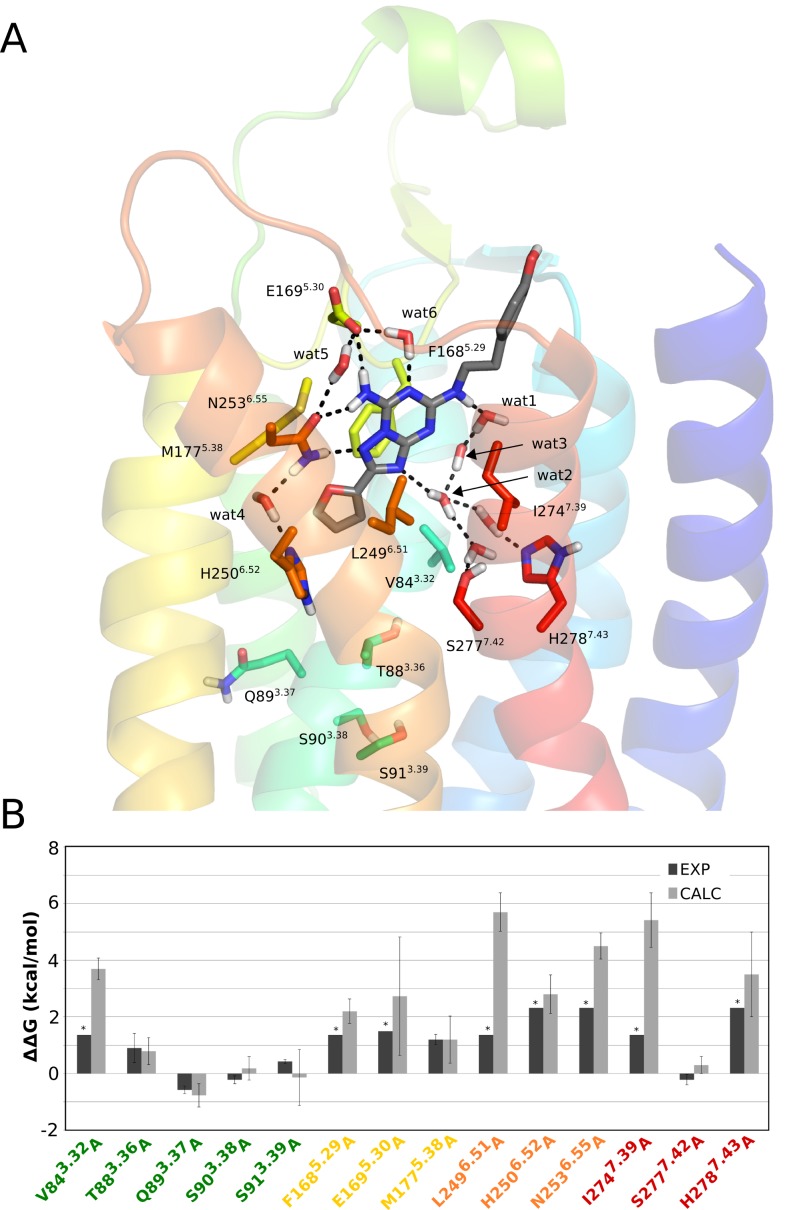
Structure of the A_2A_AR−ZM241385 complex and relative ligand binding free energies for mutants. (A) Starting structure used for the FEP simulations with TM helices shown and colored according to a rainbow representation in anti-clockwise order (TM1 = blue → TM7 = red). Residues subjected alanine mutation are depicted in sticks, together with crystal water molecules mediating receptor-ligand interactions, and dashed lines indicate hydrogen bonds. (B) Calculated (gray bars) and experimental (black bars) relative binding free energies (kcal/mol) for ZM241385 to the fourteen A_2A_AR alanine mutants compared to the wt receptor. The star symbol denotes that an experimental value could not be determined and approximates the detection threshold of the experiment.

The effect of point mutations on A_2A_AR-antagonist binding was characterized here with the triazolotriazine derivative ZM241385, a reference antagonist for which abundant experimental data is available for the purpose of this study. It is a high affinity ligand, with low nanomolar affinity for the human receptor and there are five crystal structures of hA_2A_AR in complex with ZM241385 [5], [6], [8], [9], as well as alanine scanning data for fourteen residue positions [25], [27]–[30] (these are highlighted in bold face below). The furan moiety of ZM241385 is accommodated by the deep binding pocket defined by L85^3.33^, **M177^5.38^**, **H250^6.52^** and W246^6.48^ (Figure 2). The triazolotriazine core is centrally located in the orthosteric binding pocket and overlaps with the position of the adenine core of agonists [10], [11] (Fig. 1C). This pocket is defined by the hydrophobic cleft between **F168^5.29^**, **L249^6.51^** and **I274^7.39^** together with direct hydrogen bonding to **N253^6.55^** and **E169^5.29^**.

The high resolution structure also allows the identification of both residue-residue receptor interactions and water-mediated interactions that may contribute to the ligand binding affinity. Thus, **E169^5.29^** is linked to the positively charged H264^7.29^ through a salt-bridge interaction as well as to **N253^6.55^** through a water-mediated hydrogen bond, where the latter residue is also connected to **H250^6.52^** through another water-mediated hydrogen-bond (Figure 2). The solvent containing region corresponding to the ribose binding site of the agonists, i.e. between TM1 and TM2, creates a structured water network connecting the triazolotriazine moiety with the polar residues **S277^7.42^** and **H278^7.43^**. Finally, the 4-hydroxyphenyl group of ZM241385 is partly solvent exposed and aligned with the extracellular tip of TM7. According to the electron density maps in the five crystal structures [5], [6], [8], [9] and the higher B-factors as compared to the other two moieties, it seems clear that the 4-hydroxyphenyl group has a greater flexibility in the binding site.

Ten out of the fourteen alanine mutants characterized for ZM241385 binding are involved in direct interactions (V84^3.32^A, F168^5.29^A, E169^5.30^A, M177^5.38^A, L249^6.51^A, H250^6.52^A, N253^6.55^A, I274^7.39^A) or water mediated hydrogen bonds (S277^7.42^A and H278^7.43^A) with the ligand. In addition, there is data for ZM241385 binding for four alanine mutants of positions that lie at the bottom of the binding site (T88^3.36^A, Q89^3.37^A, S90^3.38^A, S91^3.39^A). These fourteen mutants can also be classified according to their experimental effect on binding of this ligand. Eight mutants (V84^3.32^A, F168^5.29^A, E169^5.30^A, L249^6.51^A, H250^6.52^A, N253^6.55^A, I274^7.39^A and H278^7.43^A) result in undetectable radioligand binding, two mutants have a moderate effect (T88^3.36^A and M177^5.38^A) on ZM241385 affinity (between 8 and 4.6 fold), three mutants (S90^3.38^A and S91^3.39^A and S277^7.42^A) do not have any impact on antagonist binding, while one mutant (Q89^3.37^A) increases the ZM241385 binding affinity by up to 3-fold (Table 1) [25], [27]–[30].

**Figure 3:**
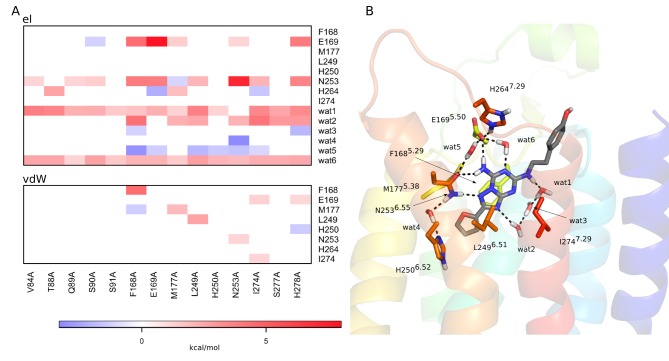
Correlation of ligand interaction energies with alanine mutations for the A_2A_AR−ZM241385 complex. (A) Correlation diagram showing the change in non-bonded interaction energies (electrostatic – top, van der Waals – bottom) for relevant binding site residues (y-axis) upon a given alanine mutation (x-axis). Only residues with any absolute interaction energy change above 1 kcal/mol are shown, where the water molecules are present throughout the MD simulations and also observed in the crystal structure. (B) The corresponding affected residues and water molecules are shown in sticks for the initial 3D structure of the complex.

Our calculations show excellent correlation with this experimental data (Figure 2). The eight mutations that display undetectable radioligand binding are all predicted to have a strong impact on the binding energetics of ZM241383, with 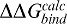 between 2.2 and 5.7 kcal/mol (Table 1). It should be noted that, except for three mutations (F168^5.29^A, M177^5.38^A and L249^6.51^A) the radioligand used in the experiments was the xanthine amine congener (XAC, see Figure 1 and Table 1). The low-resolution crystal structure of the A_2A_-XAC complex, however, confirms a clear overlay of the xanthine and triazolotriazine cores [6]. Hence, the fact that our calculations on ZM241385 show at least a 100-fold loss of affinity for all mutations with non-detectable radioligand binding is in line with this similarity in binding modes. It is noteworthy that mutations of the hydrophobic residues forming the pocket for the triazolotriazine core (V84^3.32^A, F168^5.29^A, L249^6.51^A and I274^7.39^A) cause dramatic changes in the shape of the binding pocket and as a consequence the ligand loses its main polar contacts with the receptor. This is reflected by significant reductions in the predicted binding affinities. In particular, interactions with E169^5.30^ and N253^6.55^ are severely weakened along with the direct loss of van der Waals interactions between the ligand and the mutated residue. This is clearly evident from Figure 3, where a correlation map shows the effect of mutating a given residue on the ligand interaction energetics of other residues.

The critical role of polar interactions with the ligand are also correctly captured by our calculations and mutations of E169^5.30^, H250^6.52^, N253^6.55^ and H278^7.43^ are all predicted to yield significantly reduced binding affinities. Experimentally, none of these alanine mutations show any detectable binding [28], [29]. The ZM241385 hydrogen bonds to E169^5.30^ and N253^6.55^ are thus essential for defining the ligand binding mode, together with hydrogen bonds involving structured water networks where the two histidine residues play key roles (Figure 2). These water-mediated interactions involve (crystallographically observed) stable water molecules that are present throughout all wt MD simulations. As might be expected, some of these water-mediated interactions are lost when removing surrounding hydrophobic sidechains. Particularly important are two crystal water molecules located at the bottom of the binding site, denoted wat1 and wat2 (wat2524 and wat2521, respectively, in 4EIY [9]) and an additional water molecule revealed by the simulations (wat6), which bridges the interaction between the triazoloquinazoline core and the sidechain of E169^5.30^ (Figure 2). Further, M177^5.38^ can be seen in the MD simulations to act as a hydrophobic lid, which covers the furan ring of the ligand and prevents solvent access from the extracellular cavity. Consequently, the M177^5.38^A mutation allows solvent protrusion into this region, which distorts the ligand-receptor interactions with E169^5.30^ (Figure 3).

We also explored three mutants that are located deeper in the TM cavity. The MD/FEP simulations of T88^3.36^A reproduce its rather moderate influence on ZM241385 binding (<1 kcal/mol). This effect is again mainly due to disruption of the water network established in the deeper part of the binding site and, interestingly, it is only observed for one of the two rotamers present in the high resolution crystal structure [9]. This rotamer is also the one present in all other crystal structures of this particular complex [5], [6], [8], demonstrating the power of the MD/FEP approach in eliciting the correct ligand-protein complex geometry. The S90^3.38^A and S91^3.39^A mutations in the same helix have negligible effects on ligand binding, which is also correctly predicted by the simulations. These mutations are indeed far from the binding crevice and their effects could only be assessed with the larger (34 Å radius) simulation system. The other weakly responsive mutations, Q89^3.37^A and S277^7.42^A, are also well described by the calculations, where even the slight increase in binding affinity for Q89^3.37^A is correctly captured. The calculated effect of the S277^7.42^A mutation on ZM241385 binding should be regarded as a prediction, as its measured effect was obtained with the related ligand CGS15943, as well as with the xanthine ligand XAC [29].

### Binding of the agonist NECA

**Figure 4:**
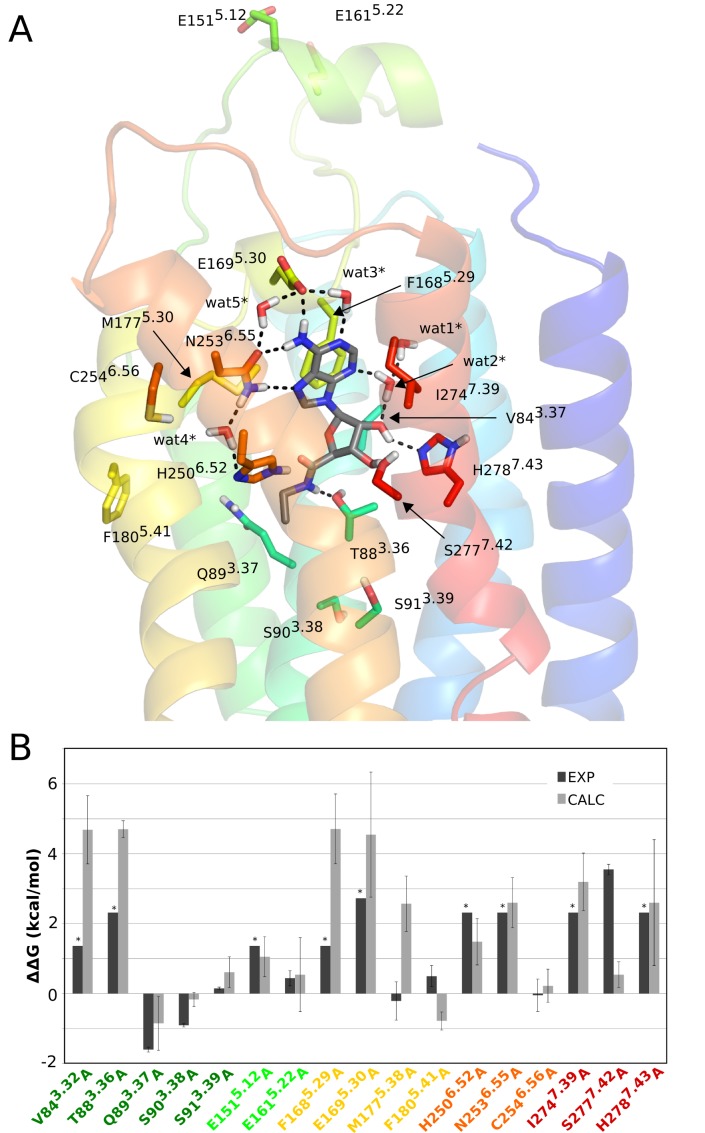
Structure of the A_2A_AR*−NECA complex and relative binding free energies for mutants. (A) Starting structure used for the FEP simulations, with explicit representation of residues subjected to alanine mutation and crystal water molecules as in Figure 2. (B) Calculated (light gray bars) and experimental (black bars) relative binding free energies (kcal/mol) for the seventeen A_2A_AR^*^ alanine mutants compared to the wt receptor. The star symbol denotes that an experimental value could not be determined and approximates the detection threshold of the experiment.

Typically agonists of the adenosine receptor family are derivatives of adenosine, where substitutions are generally tolerated in the adenine core (e.g., at positions C2 and N6) and/or in the ribose ring (e.g., at the 5′ position), while still preserving full agonist efficacy. The binding contacts of this general scaffold have been well characterized in the three crystal structures of A_2A_AR* in complex with adenosine [10], its derivative N-ethylcarboxamide adenosine (NECA) [10] and the superagonist UK432097 [11]. In addition, there is abundant mutagenesis data for the agonist NECA [25], [27]–[30] which we address here by means of MD/FEP calculations, taking advantage of the available structural information. The adenine moiety of NECA occupies the same site as the triazolotriazine moiety of ZM241385 (Figure 1C). NECA makes hydrophobic contacts with L85^3.33^, **F168^5.29^**, **L249^6.51^**, W247^6.49^, **M177^5.38^** and **I274^7.39^** and hydrogen bonds to **E169^5.30^** and **N253^6.55^** (alanine scanning results are available for residues in bold face, see Figure 4). The ribose ring is located deep in the transmembrane region, which corresponds to the highly hydrated region of the antagonist structure discussed above (Figure 1C). Two direct hydrogen bonds are established between O2′ and **H278^7.43^** and between O3′ and **S277^7.42^** while a water molecule bridges an internal hydrogen bond between O2′ and N3 of the adenine ring. The carboxamide group is anchored by two hydrogen bonds with **T88^3.36^** and **H250^6.52^**. Several structural water molecules in the binding site are observed in both inactive and active-like structures and they are also present throughout the MD simulations (Figure 4).

For NECA binding the alanine scanning data can be classified as follows (Table 2). Eight mutations showed undetectable radioligand binding (V84^3.32^A, E151^5.12^A, F168^5.29^A, E169^5.30^A, H250^6.52^A, N253^6.55^A, I274^7.39^A, H278^7.43^A), while T88^3.36^A and S277^7.42^A displayed similarly large effects on NECA binding (100- and 400-fold, respectively). Most of these residues (with the exception of E151^5.12^) have direct contacts with NECA in the crystal structure [10]. The loss of detectable radioligand binding with the agonist CGS21680 is a strong indication of a similar effect with NECA, since CGS21680 is a C2-substituted derivative of it (Figure 1B). It has also been confirmed by recent crystallographic structures that the adenine and ribose moieties of both agonists make the same ligand-receptor interactions (G. Lebón, personal communication), in line with previous agonist-bound A_2A_AR* structures [10], [11]. Among the other mutations, S91^3.39^A, E161^5.22^A, M177^5.38^A, F180^5.41^A and C254^6.56^A do not affect the binding of NECA, while Q89^3.37^A and S90^3.38^A both result in a slightly increased NECA binding affinity.

**Figure 5:**
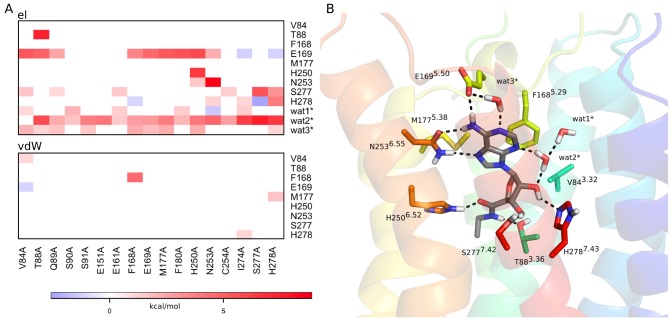
Effect of mutating a residue in the residue-ligand interaction energies for the A_2A_AR−NECA complex. (A) Correlation diagram showing the change in non-bonded interaction energies (electrostatic – top, van der Waals – bottom) for relevant binding site residues (y-axis) upon a given alanine mutation (x-axis). Only residues with any absolute interaction energy change above 1 kcal/mol are shown, where the water molecules are present throughout the MD simulations and also observed in the crystal structure. (B) The corresponding affected residues and water molecules are shown in sticks for the initial 3D structure of the complex.

The calculations are generally in good agreement with the experimental data. For most of the mutations that abolish agonist binding the estimated loss of NECA binding free energy is above the experimental detection threshold for radioligand binding (Table 2). Only in three of these cases, E151^5.12^A, H250^6.52^A and S277^7.42^A, is the estimated loss of affinity smaller than indicated by the experiments. Here, E151^5.12^ is an acidic residue far from the binding site, located on the solvent exposed face of EL2, and this mutation has been proposed to have an indirect structural effect on ligand recognition [28]. Hence, while this mutation might affect the conformation or fold of EL2 in a way that would require much longer time scale MD simulations for convergence, it should be emphasized that the experimental binding threshold of 1.4 kcal/mol actually agrees with the predicted value of 1.1 kcal/mol within our statistical error bars of ±0.6 kcal/mol. For the H250^6.52^A mutant, a hydrogen bond interaction is lost with the carbonyl group of the carboxamide moiety and the interactions with neighboring polar sidechains (E169^5.30^ and N253^6.55^) are also distorted (Figure 5). This is reflected by a 1.5 kcal/mol loss in calculated binding free energy for this ligand, which is near the detection limit of the related radiolabeled agonist CGS21680 (Table 2).

The most conspicuous result from the MD/FEP calculations on NECA binding is for the S277^7.42^A mutation. Here, the calculations predict only a moderate loss of affinity (0.5 kcal/mol) while the experimental data shows a large effect (Table 2). Since the S277^7.42^A mutation effect on antagonist binding is perfectly reproduced by the calculations (Table 1) this may point to some problem with the active-like receptor conformation (A_2A_AR*) not being fully activated, as it lacks the intracellular G-protein. The S277^7.42^A mutation clearly causes a loss of hydrogen bonding to the ribose O3′ but the simulations of the A_2A_AR* structure predict that a compensatory interaction with H278^7.43^ can be formed (Figure 5). A distinct possibility with regard to the S277^7.42^A mutation is thus that the conformation of H278^7.43^ actually changes in a manner that is not correctly captured by the simulations and that no compensatory interaction with this residue should be formed in the fully active state. That the interaction requirements with the adenosine hydroxyl groups are subtle is reflected by the fact that both 2′ and 3′-hydroxyl groups are needed for receptor activation [46], while removal of only one of the two hydroxyl groups leads to partial agonism [47].

It should also be noted that the displaced radioligand in the S277^7.42^A case is an antagonist (XAC), which raises the general question of the contribution of the inactive *vs.* active apo-receptor equilibrium free energy to the measured *K*_i_ value. Note that the displacement of an antagonist by an agonist, and vice versa, will always contain this contribution and may therefore not be optimal for measuring binding affinity to the desired receptor state. In case of the S277^7.42^A mutation, the predicted loss of agonist binding affinity to the active state would indeed be smaller than indicated by the experimental measurement if the mutation shifts the apo-receptor equilibrium towards the inactive state. However, the simulation results for NECA binding to the T88^3.36^A mutant are in better agreement with the experimental data, which were also measured with the XAC antagonist. Perhaps most important from a structural viewpoint, in the T88^3.36^A case there are no compensatory polar residues in the vicinity of the mutated sidechain, which explains why the effect of this mutation on NECA binding may be less sensitive to the exact protein conformation.

**Figure 6:**
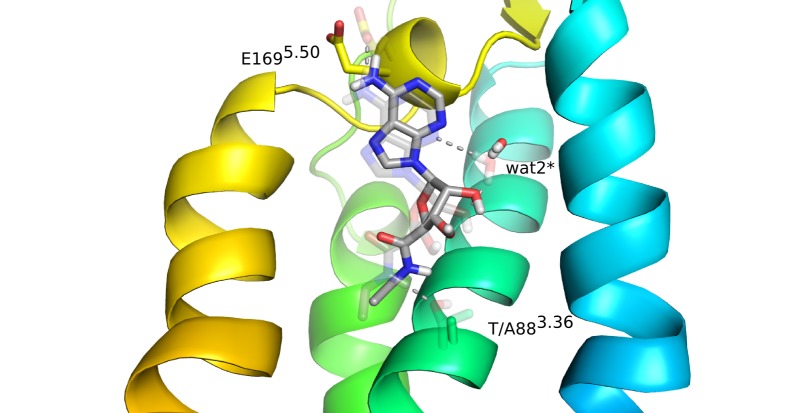
Structural effects of the T88^3.36^A mutation on agonist binding. The average MD conformation of the T88^3.36^A mutant NECA complex with interacting sidechains and water molecules shown (solid sticks) overlaid on the initial wt conformation (transparent sticks).

The analysis of the indirect effects of mutations in Figure 5 interestingly shows how interaction networks are coupled in the receptor. Thus, while the T88^3.36^A mutant removes an anchoring hydrogen bond to the carboxamide of NECA, it also destabilizes the ligand in such a way that it loses key polar contacts with E169^5.30^ and wat2* (denoted w2027 in 2YDV) as shown in Figure 6. It is also noteworthy that the favorable effect of Q89^3.37^A on NECA binding is seen both experimentally and in our calculations. Here, the slight worsening of certain ligand-receptor interactions (Figure 5) is counterbalanced by the overall beneficial effect on the protein conformation achieved by the alanine mutant, in agreement with the effect on thermal stability observed for this mutation [48]. The remaining mutations are not in the binding site, but either deeper in the TM cavity (S90^3.38^, S91^3.39^), facing the membrane bilayer (F180^5.41^, C254^6.56^) or water exposed (E161^5.22^), and mutating them to alanine does not have any impact on the binding affinity of the ligand.

Finally, the apparent discrepancy of the M177^5.38^A mutation should be noted. Here, while the experimental data for NECA indicates no effect on agonist binding, the binding of the closely related agonist CGS21680 (Figure 1) was significantly affected [25]. This is particularly strange since the two compounds are identical in the binding region near M177^5.38^ (Figure 4A). The MD/FEP simulations are thus in better agreement with the latter observation and also with the binding affinity estimates reported [25], indicating that M177^5.38^ could play the same hydrophobic role in agonist binding as previously described for antagonist binding. To further examine this hypothesis, we performed additional simulations of the M177^5.38^A mutation to evaluate its predicted effect on the CGS21680 agonist binding affinity, i.e., with this ligand docked into the receptor site. As expected, the result (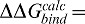
1.5±0.9 kcal/mol) is in good agreement with the experimental data for this ligand (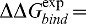
11.2±0.3 kcal/mol). As discussed above, this again indicates that there may be a problem with the experimental estimate using an antagonist radioligand (ZM2341385) to measure agonist affinity as mutational effects on the apo-receptor equilibrium again cannot be excluded.

## Discussion

The prospect of accurate computational prediction of site-directed mutagenesis experiments is of great interest in biochemistry and pharmacology. Structural investigations of ligand binding to GPCRs have traditionally been guided by a combination of molecular modeling and site-directed mutagenesis [49], but with the recent availability of crystal structures it has become possible to re-examine and interpret the existing experimental data at another level of detail. This will also allow the design of mutations to examine the binding of new ligands and, in this context, accurate methods will be needed for predicting binding free energies of ligands to wild-type and mutated receptors. The free energy calculation scheme used herein has demonstrated an improvement in convergence, precision and accuracy as compared with standard FEP protocols [31]. The technique was earlier used to validate a homology-based model of the Y1 neuropeptide receptor and to discriminate against suboptimal ligand docking models of the same complex. Here, we have applied the same scheme for agonist (NECA) and antagonist (ZM241385) binding to A_2A_AR, two complexes that have been crystallized with this receptor, thus avoiding the uncertainties inherent in homology modeling and ligand docking. Moreover, the high resolution of the crystal structures in combination with MD simulations allow the role of structural water molecules in ligand binding to be assessed, which is a clear advantage over previous computational analyses of mutagenesis data for the same system [25].

With regard to the applicability of molecular dynamics free energy calculations, it should also be mentioned that *in silico* mutations have recently been reported for the A_2A_ receptor, where the goal was to correlate calculated free energies with thermostability [50]. However, besides the use of a suboptimal free energy perturbation (thermodynamic integration) protocol where entire residues are mutated in one go, there appear to be other problems with that study as well. First, there is no indication that a proper thermodynamic cycle was used, which is absolutely necessary in order to address thermostability. That is, for every residue mutation in the folded receptor (active or inactive) the corresponding mutation has to be done for a model of the unfolded state. For example, in early work on thermostability a tripeptide was used as the reference unfolded state [51]. Second, mutations of charged residues are not trivial to handle in free energy calculations, as the net charge of the system changes [31]. In [50] removal of positive arginine and lysine charges yielded calculated free energies on the order of only 10 kcal/mol and negative, which seems to indicate a problem with electrostatics. In the present case, where we study the effect of single point mutants on ligand binding, the reference state (i.e., apo simulations) is explicitly considered in our free energy scheme and thus the thermodynamic cycle is derived from the two parallel simulations of the receptor, i.e. in the holo and apo states [31].

Jaakola *et al.* have also evaluated agonist and antagonist binding to some A_2A_AR mutants by considering static A_2A_AR and A_2A_AR* models, using a scoring function-like method implemented in the ICM software [25]. Despite reasonable correlation with experimental data that approach is, however, by definition insensitive to the effect of water-mediated interactions, as recognized by the authors. One of the mutants evaluated in that study, M177^5.38^A, was predicted to have a moderate impact on both agonist and antagonist binding but the interpretation remained unclear. Our results show that this mutant causes the hydrophobic cleft between TM5 and TM6 to become hydrated, thereby disturbing both interhelical and receptor-ligand interactions.

In general, the interpretation of site-directed mutagenesis experiments such as alanine scanning is far from trivial. The change in ligand binding affinity between wt and mutated receptors is estimated on the basis of radioligand competition assays. Thus, the given ligand must displace the reference radioligand in both wt and mutant receptors in order for a *K*_i_ value to be measured. This is a limitation in those cases where the mutation completely abolishes radioligand binding, in which case only a qualitative estimate can be obtained, provided that the competing ligand is chemically similar to the radioligand or at least occupies the same binding cavity. This point is nicely illustrated by the S277^7.42^A mutant [29]. No radioligand binding was detected for [H^3^]NECA with this mutation, thus leading to an estimated effect of >2.3 kcal/mol on the affinity of this ligand. However, the same mutant displayed sufficient affinity for another radioligand ([H^3^]XAC) which allowed a more precise assessment of the effect on NECA binding affinity (3.5±0.2 kcal/mol) based on a competition assay. Since this is not always possible to achieve, the lack of affinity for available radioligands can in many cases preclude quantitative estimation of mutation effects on ligand binding. We have seventeen such cases in our dataset, eight mutants in the A_2A_AR-ZM241385 system and nine mutants in A_2A_AR*-NECA, and it is noteworthy that our predictions show a numerical value that in most cases is higher than the detection threshold in the experiments (marked with stars in Figures 2 and 4). Moreover, in thirteen out of the seventeen cases, our calculations should be considered as predictions of similarly significant effects on NECA or ZM241385 binding, given that the radioligand used was a different one. It may be useful to point out that an extensive review of the combinations of radioligands, competition ligands and mutations is provided in [26] and this information is continuously updated in the GPCRDB database [45].

Our simulation results also show a good correlation with the experimental data for moderate (both positive and negative) and negligible effects on the binding affinities. In addition, the calculations yield new structure-based information on ligand binding to some mutants, particularly for the antagonist ZM241385. Many of the experiments were performed before the tritiated version of this ligand was available [52], and the precise effect on this reference ligand could not be measured for many mutants. Thus, the strong impact on ZM241385 binding was not evident for some mutants, such as H278^7.43^A. This residue appears to play a key role in maintaining a structural water network, which occupies an equivalent region to the ribose binding site in the A_2A_AR* structures (Figure 2). This water network has, in fact, been highlighted by several computational studies on antagonist binding [15], [17], but its relation to mutagenesis data has not been addressed before.

Apart from this water network in antagonist binding, which is displaced by the ribose moiety of the agonists, there are several structural water molecules revealed by the crystal structures that could potentially play a role in ligand binding. Some of them are common to the A_2A_ and A_2A_AR* structures, such as the water molecules connecting N253^6.55^ with H250^6.52^ and N253^6.55^ with E169^5.30^ (wat4/wat4* and wat5/wat5*, respectively, in Figures 2 and 4). These are also observed in the corresponding crystal structures (4EIY and 2YDO). The former was found to be particularly important in defining the observed conformation of N253^6.55^ and indeed, if removed from the initial structure, the calculations show insensitivity to the N253^6.55^A mutation. This clearly supports the indirect role of this water molecule (wat4) in ligand binding. In the case of agonist binding, water molecules are particularly important in maintaining the polar interactions with the ribose as recognized previously [10], [53]. Our simulations show a great stability of several water molecules in contact with the ribose and particularly wat2* (also coordinated to wat1*) is essential for maintaining the ribose bioactive conformation (Figure 5). In general, as can be seen from Figures 3 and 5, the overall loss of favourable ligand-water interactions for mutations is correlated with losses in ligand binding affinity. Hence, it appears that the role of water-mediated interactions is of major importance for this receptor system, and they should consequently be carefully considered in any ligand design project.

## Conclusions

The effects of point mutations on ligand binding constitute an important information resource for characterization of protein function and protein-ligand interactions, with obvious applications to pharmacology. Accurate and fast methodologies to predict the structural and energetic effects of a given mutation on ligand binding are useful not only for design and prediction of site-directed mutagenesis experiments, but also for characterization of genetic variations associated with individual responses to drug treatment. This work provides a distinct validation of our computational strategy, based on MD/FEP simulations, to quantitatively assess the effects of point-mutations on ligand binding. The methodology has been applied to successfully analyze the effects of alanine scanning on two different GPCRs (the A_2A_ and NPY1 receptors [31]) with ligands of different chemical nature. It is also straightforward to apply this FEP scheme to series of chemically similar ligands, as demonstrated earlier [31]. The basic principle to use a series of successive subperturbations in the calculations to get a smoother transformation between the end-states, yields improved convergence, precision and accuracy. Moreover, the use of all-atom MD simulations is important for understanding the energetic effects of key interactions mediated by water molecules, in addition to the direct protein-ligand interactions. This is likely to be of conservable importance both for the design and interpretation of mutagenesis data and in ligand design projects.

## Supporting Information

Table S1 **Calculated and experimental ZM241385 relative binding free energies for A_2A_AR mutants using different simulation sphere sizes.**
(DOCX)

Table S2 **Calculated and experimental NECA relative binding free energies for A_2A_AR mutants using different simulation sphere sizes.**
(DOCX)

## References

[1] Hopkins AL, Groom CR (2002) The druggable genome. Nat Rev Drug Discov 1: 727–730. 12209152 10.1038/nrd892

[2] Venkatakrishnan AJ, Deupi X, Lebon G, Tate CG, Schertler GF, et al (2013) Molecular signatures of G-protein-coupled receptors. Nature 494: 185–194. 23407534 10.1038/nature11896

[3] Gutiérrez-de-Terán H (2014) The roles of computational chemistry in the ligand design of G protein-coupled receptors: how far have we come and what should we expect? Future Med Chem 6: 251–254. 24575961 10.4155/fmc.13.209

[4] Salon JA, Lodowski DT, Palczewski K (2011) The significance of G protein-coupled receptor crystallography for drug discovery. Pharmacol Rev 63: 901–937. 21969326 10.1124/pr.110.003350 PMC3186081

[5] Jaakola V-P, Griffith MT, Hanson MA, Cherezov V, Chien EYT, et al (2008) The 2.6 Angstrom Crystal Structure of a Human A2A Adenosine Receptor Bound to an Antagonist. Science 322: 1211–1217. 18832607 10.1126/science.1164772 PMC2586971

[6] Doré AS, Robertson N, Errey JC, Ng I, Hollenstein K, et al (2011) Structure of the Adenosine A2A Receptor in Complex with ZM241385 and the Xanthines XAC and Caffeine. Structure 19: 1283–1293. 21885291 10.1016/j.str.2011.06.014 PMC3732996

[7] Congreve M, Andrews SP, Doré AS, Hollenstein K, Hurrell E, et al (2012) Discovery of 1,2,4-triazine derivatives as adenosine A(2A) antagonists using structure based drug design. J Med Chem 55: 1898–1903. 22220592 10.1021/jm201376w PMC3308197

[8] Hino T, Arakawa T, Iwanari H, Yurugi-Kobayashi T, Ikeda-Suno C, et al (2012) G-protein-coupled receptor inactivation by an allosteric inverse-agonist antibody. Nature 482: 237–240. 22286059 10.1038/nature10750 PMC3303121

[9] Liu W, Chun E, Thompson AA, Chubukov P, Xu F, et al (2012) Structural Basis for Allosteric Regulation of GPCRs by Sodium Ions. Science 337: 232–236. 22798613 10.1126/science.1219218 PMC3399762

[10] Lebon G, Warne T, Edwards PC, Bennett K, Langmead CJ, et al (2011) Agonist-bound adenosine A2A receptor structures reveal common features of GPCR activation. Nature 474: 521–525. 21593763 10.1038/nature10136 PMC3146096

[11] Xu F, Wu H, Katritch V, Han GW, Jacobson KA, et al (2011) Structure of an Agonist-Bound Human A2A Adenosine Receptor. Science 332: 322–327. 21393508 10.1126/science.1202793 PMC3086811

[12] Rasmussen SGF, Choi H-J, Fung JJ, Pardon E, Casarosa P, et al (2011) Structure of a nanobody-stabilized active state of the β(2) adrenoceptor. Nature 469: 175–180. 21228869 10.1038/nature09648 PMC3058308

[13] Park JH, Scheerer P, Hofmann KP, Choe H-W, Ernst OP (2008) Crystal structure of the ligand-free G-protein-coupled receptor opsin. Nature 454: 183–187. 18563085 10.1038/nature07063

[14] Bacilieri M, Ciancetta A, Paoletta S, Federico S, Cosconati S, et al (2013) Revisiting a Receptor-Based Pharmacophore Hypothesis for Human A 2AAdenosine Receptor Antagonists. J Chem Inf Model 53: 1620–1637. 23705857 10.1021/ci300615u

[15] Bortolato A, Tehan BG, Bodnarchuk MS, Essex JW, Mason JS (2013) Water Network Perturbation in Ligand Binding: Adenosine A 2AAntagonists as a Case Study. J Chem Inf Model 53: 1700–1713. 23725291 10.1021/ci4001458

[16] Sabbadin D, Ciancetta A, Moro S (2014) Bridging Molecular Docking to Membrane Molecular Dynamics To Investigate GPCR–Ligand Recognition: The Human A2A Adenosine Receptor as a Key Study. J Chem Inf Model 54: 169–183. 24359090 10.1021/ci400532b

[17] Higgs C, Beuming T, Sherman W (2010) Hydration Site Thermodynamics Explain SARs for Triazolylpurines Analogues Binding to the A2A Receptor. ACS Med Chem Lett 1: 160–164. 24900189 10.1021/ml100008s PMC4007955

[18] Ng HW, Laughton CA, Doughty SW (2013) Molecular Dynamics Simulations of the Adenosine A2a Receptor: Structural Stability, Sampling, and Convergence. J Chem Inf Model 53: 1168–1178. 23514445 10.1021/ci300610w

[19] Carlsson J, Yoo L, Gao Z-G, Irwin JJ, Shoichet BK, et al (2010) Structure-based discovery of A2A adenosine receptor ligands. J Med Chem 53: 3748–3755. 20405927 10.1021/jm100240h PMC2865168

[20] Katritch V, Jaakola V-P, Lane JR, Lin J, Ijzerman AP, et al (2010) Structure-based discovery of novel chemotypes for adenosine A(2A) receptor antagonists. J Med Chem 53: 1799–1809. 20095623 10.1021/jm901647p PMC2826142

[21] van der Horst E, van der Pijl R, Mulder-Krieger T, Bender A, Ijzerman AP (2011) Substructure-based virtual screening for adenosine A2A receptor ligands. ChemMedChem 6: 2302–2311. 22021213 10.1002/cmdc.201100369

[22] Langmead CJ, Andrews SP, Congreve M, Errey JC, Hurrell E, et al (2012) Identification of Novel Adenosine A 2AReceptor Antagonists by Virtual Screening. J Med Chem 55: 1904–1909. 22250781 10.1021/jm201455y PMC3308209

[23] Chen D, Ranganathan A, Ijzerman AP, Siegal G, Carlsson J (2013) Complementarity between in Silico and Biophysical Screening Approaches in Fragment-Based Lead Discovery against the A 2AAdenosine Receptor. J Chem Inf Model 53: 2701–2714. 23971943 10.1021/ci4003156

[24] Tosh DK, Phan K, Gao Z-G, Gakh AA, Xu F, et al (2012) Optimization of Adenosine 5′-Carboxamide Derivatives as Adenosine Receptor Agonists Using Structure-Based Ligand Design and Fragment Screening. J Med Chem 55: 4297–4308. 22486652 10.1021/jm300095s PMC3479662

[25] Jaakola VP, Lane JR, Lin JY, Katritch V, Ijzerman AP, et al (2010) Ligand Binding and Subtype Selectivity of the Human A2A Adenosine Receptor: Identification and characterization of essential amino acid residues. J Biol Chem 285: 13032–13044. 20147292 10.1074/jbc.M109.096974 PMC2857108

[26] Martinelli A, Tuccinardi T (2008) Molecular modeling of adenosine receptors: new results and trends. Med Res Rev 28: 247–277. 17492754 10.1002/med.20106

[27] Jiang Q, Van Rhee AM, Kim J, Yehle S, Wess J, et al (1996) Hydrophilic side chains in the third and seventh transmembrane helical domains of human A2A adenosine receptors are required for ligand recognition. Mol Pharmacol 50: 512–521. 8794889 PMC3418326

[28] Kim J, Jiang Q, Glashofer M, Yehle S, Wess J, et al (1996) Glutamate residues in the second extracellular loop of the human A2a adenosine receptor are required for ligand recognition. Mol Pharmacol 49: 683–691. 8609897 PMC3425639

[29] Kim J, Wess J, Van Rhee AM, Schöneberg T, Jacobson KA (1995) Site-directed mutagenesis identifies residues involved in ligand recognition in the human A2a adenosine receptor. J Biol Chem 270: 13987–13997. 7775460 10.1074/jbc.270.23.13987 PMC3427751

[30] Jiang Q, Lee BX, Glashofer M, van Rhee AM, Jacobson KA (1997) Mutagenesis Reveals Structure−Activity Parallels between Human A 2AAdenosine Receptors and Biogenic Amine G Protein-Coupled Receptors. J Med Chem 40: 2588–2595. 9258366 10.1021/jm970084v PMC3449164

[31] Boukharta L, Gutiérrez-de-Terán H, Aqvist J (2014) Computational prediction of alanine scanning and ligand binding energetics in G-protein coupled receptors. PLoS Comput Biol 10: e1003585. 24743773 10.1371/journal.pcbi.1003585 PMC3990513

[32] Gutiérrez-de-Terán H, Massink A, Rodríguez D, Liu W, Han GW, et al (2013) The Role of a Sodium Ion Binding Site in the Allosteric Modulation of the A2A Adenosine G Protein-Coupled Receptor. Structure 21: 2175–2185. 24210756 10.1016/j.str.2013.09.020 PMC3858454

[33] Ballesteros JA, Weinstein H (1995) Integrated methods for the construction of three-dimensional models and computational probing of structure-function relations in G protein-coupled receptors. Methods Neurosci 25: 366–428.

[34] Gutiérrez-de-Terán H, Bello X, Rodríguez D (2013) Characterization of the dynamic events of GPCRs by automated computational simulations. Biochem Soc Trans 41: 205–212. 23356284 10.1042/BST20120287

[35] Hess B, Kutzner C, van der Spoel D, Lindahl E (2008) GROMACS 4: Algorithms for Highly Efficient, Load-Balanced, and Scalable Molecular Simulation. J Chem Theory Comput 4: 435–447. 26620784 10.1021/ct700301q

[36] Rodríguez D, Piñeiro A, Gutiérrez-de-Terán H (2011) Molecular Dynamics Simulations Reveal Insights into Key Structural Elements of Adenosine Receptors. Biochemistry 50: 4194–4208. 21480628 10.1021/bi200100t

[37] Jorgensen WL, Maxwell DS, Tirado-Rives J (1996) Development and Testing of the OPLS All-Atom Force Field on Conformational Energetics and Properties of Organic Liquids. J Am Chem Soc 118: 11225–11236.

[38] Kaminski GA, Friesner RA, Tirado-Rives J, Jorgensen WL (2001) Evaluation and reparametrization of the OPLS-AA force field for proteins via comparison with accurate quantum chemical calculations on peptides. J Phys Chem B 105: 6474–6487.

[39] ffld_server, Schrodinger, 2011. Available: http://www.schrodinger.com.

[40] Berger O, Edholm O, Jähnig F (1997) Molecular dynamics simulations of a fluid bilayer of dipalmitoylphosphatidylcholine at full hydration, constant pressure, and constant temperature. Biophys J 72: 2002–2013. 9129804 10.1016/S0006-3495(97)78845-3 PMC1184396

[41] Marelius J, Kolmodin K, Feierberg I, Aqvist J (1998) Q: a molecular dynamics program for free energy calculations and empirical valence bond simulations in biomolecular systems. J Mol Graph Model 16: 213–225. 10522241 10.1016/s1093-3263(98)80006-5

[42] King G, Warshel A (1989) A surface constrained all-atom solvent model for effective simulations of polar solutions. J Chem Phys 91: 3647–3661.

[43] Lee FS, Warshel A (1992) A local reaction field method for fast evaluation of long-range electrostatic interactions in molecular simulations. J Chem Phys 97: 3100.

[44] Ryckaert J-P, Ciccotti G, Berendsen HJ (1977) Numerical integration of the cartesian equations of motion of a system with constraints: molecular dynamics of n-alkanes. J Comput Phys 23: 327–341.

[45] Vroling B, Sanders M, Baakman C, Borrmann A, Verhoeven S, et al (2010) GPCRDB: information system for G protein-coupled receptors. Nucleic Acids Res 39: D309–D319. 21045054 10.1093/nar/gkq1009 PMC3013641

[46] van der Wenden EM, Frijtag Drabbe Kuenzel von JK, Mathot RAA, Danhof M, Ijzerman AP, et al (1995) Ribose-Modified Adenosine Analogs as Potential Partial Agonists for the Adenosine Receptor. J Med Chem 38: 4000–4006. 7562934 10.1021/jm00020a014

[47] Vittori S, Lorenzen A, Stannek C, Costanzi S, Volpini R, et al (2000) N-cycloalkyl derivatives of adenosine and 1-deazaadenosine as agonists and partial agonists of the A(1) adenosine receptor. J Med Chem 43: 250–260. 10649980 10.1021/jm9911231

[48] Lebon G, Bennett K, Jazayeri A, Tate CG (2011) Thermostabilisation of an Agonist-Bound Conformation of the Human Adenosine A2A Receptor. J Mol Biol 409: 298–310. 21501622 10.1016/j.jmb.2011.03.075 PMC3145977

[49] Kristiansen K (2004) Molecular mechanisms of ligand binding, signaling, and regulation within the superfamily of G-protein-coupled receptors: molecular modeling and mutagenesis approaches to receptor structure and function. Pharmacol Ther 103: 21–80. 15251227 10.1016/j.pharmthera.2004.05.002

[50] Lee S, Bhattacharya S, Grisshammer R, Tate C, Vaidehi N (2014) Dynamic Behavior of the Active and Inactive States of the Adenosine A2A Receptor. J Phys Chem B 118: 3355–3365. 24579769 10.1021/jp411618h PMC3983344

[51] Dang LX, Merz KM, Kollman PA (1989) Free energy calculations on protein stability: Thr-157 -> Val-157 mutation of T4 lysozyme. J Am Chem Soc 111: 8505–8508.

[52] Alexander SP, Millns PJ (2001) [(3)H]ZM241385–an antagonist radioligand for adenosine A(2A) receptors in rat brain. Eur J Pharmacol 411: 205–210. 11164377 10.1016/s0014-2999(00)00899-2

[53] Deflorian F, Kumar TS, Phan K, Gao Z-G, Xu F, et al (2012) Evaluation of Molecular Modeling of Agonist Binding in Light of the Crystallographic Structure of an Agonist-Bound A 2AAdenosine Receptor. J Med Chem 55: 538–552. 22104008 10.1021/jm201461q PMC3261785

