# Supplementary material for: Structural and Energetic Effects of A2A Adenosine Receptor Mutations on Agonist and Antagonist Binding
Source: PLoS One. 2014 Oct 6;9(10):e108492. doi: 10.1371/journal.pone.0108492 (PMC4186821; doi:10.1371/journal.pone.0108492)
Supplement: Table S1 — Calculated and experimental ZM241385 relative binding free energies for A2AAR mutants using different simulation sphere sizes. (DOCX) [file pone.0108492.s001.docx]

**Table S1**. Calculated and experimental ZM241385 relative binding free energies for A_2A_AR mutants using different simulation sphere sizes.

| Mutant | $\Delta\Delta G_{bind}^{\exp}$^a^ | $\Delta\Delta G_{bind}^{calc}$ 25 Å | $\Delta\Delta G_{bind}^{calc}$ 34 Å |
| --- | --- | --- | --- |
| V84^3.32^A | NB^b^ (>1.4) [30] | 3.7 ± 0.4 | 3.5 ± 1.0 |
| T88^3.36^A | 0.9 ± 0.5 [27] | 0.8 ± 0.5 | 0.0 ± 0.7 |
| Q89^3.37^A | -0.6 ± 0.1 [27] | -0.8 ± 0.4 | -0.5 ± 0.8 |
| S90^3.38^A | -0.2 ± 0.1 [27] | ND^c^ | 0.2 ± 0.4^c^ |
| S91^3.39^A | 0.4 ± 0.1 [27] | ND^c^ | -0.1 ± 1.0^c^ |
| F168^5.29^A | NB^b^ (>1.4) [25] | 2.2 ± 0.4 | 4.6 ± 0.6 |
| E169^5.30^A | NB^b^ (>1.5) [28] | 2.7 ± 1.5 | 6.1 ± 1.5 |
| M177^5.38^A | 1.2 ± 0.2 [25] | 1.2 ± 0.8 | 0.4 ± 0.7 |
| L249^6.51^A | NB^b^ (>1.4) [25] | 5.7 ± 0.7 | 6.4 ± 0.8 |
| H250^6.52^A | NB^b^ (>2.3) [29] | 2.8 ± 0.7 | 3.4 ± 1.8 |
| N253^6.55^A | NB^b^ (>2.3) [29] | 4.5 ± 0.5 | 4.6 ± 0.6 |
| I274^7.39^A | NB^b^ (>1.4) [29] | 5.4 ± 1.0 | 4.8 ± 1.0 |
| S277^7.42^A | -0.2 ± 0.2 [29](XAC)^d^  -0.1 ± 0.2 (CGS15943) | 0.3 ± 0.3 | 0.5 ± 0.5 |
| H278^7.43^A | NB (>2.3) [29] | 3.5 ± 1.5 | 0.8 ± 1.9 |

^a^ Experimental relative binding free energies ($\Delta\Delta G_{bind}^{\exp}$) calculated from *K*_i_ values as $\Delta\Delta G_{bind}^{\exp}=RT\ln(K_{i}^{mut}/K_{i}^{wt})$.

^b^ NB = non-detectable radioligand binding. The value corresponding to the experimental detection threshold is indicated within parentheses.

^c^ ND = not determined. The mutated position is outside the boundaries of the default 25 Å sphere.

^d^ Experimental data is only available for the antagonists XAC and CGS15943.
