# Supplementary material for: Structural and Energetic Effects of A2A Adenosine Receptor Mutations on Agonist and Antagonist Binding
Source: PLoS One. 2014 Oct 6;9(10):e108492. doi: 10.1371/journal.pone.0108492 (PMC4186821; doi:10.1371/journal.pone.0108492)
Supplement: Table S2 — Calculated and experimental NECA relative binding free energies for A2AAR mutants using different simulation sphere sizes. (DOCX) [file pone.0108492.s002.docx]

**Table S2**. Calculated and experimental NECA relative binding free energies for A_2A_AR mutants using different simulation sphere sizes.

| Mutant | $\Delta\Delta G_{bind}^{\exp}$^a^ | $\Delta\Delta G_{bind}^{calc}$ 25 Å | $\Delta\Delta G_{bind}^{calc}$ 34 Å |
| --- | --- | --- | --- |
| V84^3.32^A | NB^b^ (>1.4) [30] | 4.7 ± 1.0 | 2.1 ± 0.7 |
| T88^3.36^A | 2.6± 0.2 [27] | 4.7 ± 0.2 | 4.8 ± 0.6 |
| Q89^3.37^A | -1.6 ± 0.1 [27] | -0.9 ± 0.8 | 0.0 ± 1.2 |
| S90^3.38^A | -0.9 ± 0.0 [27] | -0.2 ± 0.2 | 0.0 ± 0.3 |
| S91^3.39^A | 0.2 ± 0.0 [27] | 0.6 ± 0.4 | 1.5 ± 0.4 |
| E151^5.12^A | NB^b^ (>1.4) [28] | ND^c^ | 1.1 ± 0.6 |
| E161^5.22^A | 0.4 ± 0.2 [28] | ND^c^ | 0.5 ± 1.1 |
| F168^5.29^A | NB^b^ (>1.4) [25] | 4.7 ± 1.0 | 5.7 ± 0.8 |
| E169^5.30^A | NB^b^ (>2.7) [28] | 4.5 ± 1.8 | 3.4 ± 1.6 |
| M177^5.38^A | -0.2 ± 0.5 [25] | 2.6 ± 0.8 | 2.1 ± 0.9 |
| F180^5.41^A | 0.5 ± 0.3 [29] | -0.8 ± 0.3 | -0.6 ± 0.8 |
| H250^6.52^A | NB^b^ (>2.3) [29] | 1.5 ± 0.7 | 2.5 ± 1.3 |
| N253^6.55^A | NB^b^ (>2.3) [29] | 2.6 ± 0.7 | 3.3 ± 1.2 |
| C254^6.56^A | -0.1 ± 0.5 [29] | ND^c^ | 0.2 ± 0.5 |
| I274^7.39^A | NB^b^ (>2.3) [29] | 3.2 ± 0.8 | 2.5 ± 1.1 |
| S277^7.42^A | 3.5± 0.2 [29] | 0.5 ± 0.4 | 1.1 ± 0.7 |
| H278^7.43^A | NB^b^ (>2.3)[29] | 2.6 ± 1.8 | 3.0 ± 2.3 |

^a^ Experimental relative binding free energies ($\Delta\Delta G_{bind}^{\exp}$) calculated from *K*_i_ values as $\Delta\Delta G_{bind}^{\exp}=RT\ln(K_{i}^{mut}/K_{i}^{wt})$.

^b^ NB = non-detectable radioligand binding. The value corresponding to the experimental detection threshold is indicated within parenthesis.

^c^ ND = not determined. The mutated position is outside the boundaries of the default 25 Å sphere.
